# Modeling the Dynamics of High-Grade Serous Ovarian Cancer Progression for Transvaginal Ultrasound-Based Screening and Early Detection

**DOI:** 10.1371/journal.pone.0156661

**Published:** 2016-06-03

**Authors:** Dana-Adriana Botesteanu, Jung-Min Lee, Doron Levy

**Affiliations:** 1 Department of Mathematics and Center for Scientific Computation and Mathematical Modeling (CSCAMM), University of Maryland, College Park, Maryland, United States of America; 2 Women’s Malignancies Branch, Center for Cancer Research, National Cancer Institute, Bethesda, Maryland, United States of America; University of California, Irvine, UNITED STATES

## Abstract

High-grade serous ovarian cancer (HGSOC) represents the majority of ovarian cancers and accounts for the largest proportion of deaths from the disease. A timely detection of low volume HGSOC should be the goal of any screening studies. However, numerous transvaginal ultrasound (TVU) detection-based population studies aimed at detecting low-volume disease have not yielded reduced mortality rates. A quantitative invalidation of TVU as an effective HGSOC screening strategy is a necessary next step. Herein, we propose a mathematical model for a quantitative explanation on the reported failure of TVU-based screening to improve HGSOC low-volume detectability and overall survival.We develop a novel *in silico* mathematical assessment of the efficacy of a unimodal TVU monitoring regimen as a strategy aimed at detecting low-volume HGSOC in cancer-positive cases, defined as cases for which the inception of the first malignant cell has already occurred. Our findings show that the median window of opportunity interval length for TVU monitoring and HGSOC detection is approximately 1.76 years. This does not translate into reduced mortality levels or improved detection accuracy in an *in silico* cohort across multiple TVU monitoring frequencies or detection sensitivities. We demonstrate that even a semiannual, unimodal TVU monitoring protocol is expected to miss detectable HGSOC. Lastly, we find that circa 50% of the simulated HGSOC growth curves never reach the baseline detectability threshold, and that on average, 5–7 infrequent, rate-limiting stochastic changes in the growth parameters are associated with reaching HGSOC detectability and mortality thresholds respectively. Focusing on a malignancy poorly studied in the mathematical oncology community, our model captures the dynamic, temporal evolution of HGSOC progression. Our mathematical model is consistent with recent case reports and prospective TVU screening population studies, and provides support to the empirical recommendation against frequent HGSOC screening.

## Introduction

Ovarian cancer is a relatively rare disease, representing 2.6% of all new cancer cases in US women [1]. However, ovarian cancer is the most fatal gynecologic cancer with approximately 35% five-year overall survival rate; in 2015, it is estimated that 21,290 new cases of ovarian cancer with an estimated 14,180 deaths related to this disease will occur [2]. The inability to detect aggressive, early stage ovarian cancer has substantial implications for the reported low post-diagnosis survival rates. This is possibly, in part, due to the natural history of ovarian cancers, since most women with localized disease present vague symptoms such as pelvic or abdominal pain, abdominal bloating, urinary urgency or frequency and early satiety [3]. A recently proposed morphomolecular characterization of ovarian cancers underscores the importance of clear separation between the various subtypes of ovarian cancers with respect to the appropriate future therapeutic targeting [4]; therein, it is reported that epithelial ovarian cancers account for 85–90% of ovarian cancers, with a subset of epithelial ovarian cancers, high-grade serous ovarian cancers (HGSOCs) representing nearly 70% of all ovarian cancer cases.

Focusing on HGSOC, clinical features of its progression prior to detection are difficult to observe. Only circa 15% of HGSOC are solely localized to the ovary or fallopian tubes at the time of diagnosis [1, 5] and about 35% of what is thought to be a malignant mass is actually an adnexal benign mass [6]. Moreover, the normal tubo-ovarian environment is regarded as temporally heterogeneous in both pre- and postmenopausal stages with respect to hormonal fluctuations, growth factor and reactive *O*_2_ species, making a departure from healthy homeostasis difficult to observe [7]. HGSOC causality, initiation and duration of its pre-diagnosis stage thus remain difficult to study *in vivo* or estimate *in vitro*.

Existing early detection screening strategies for other cancer types, including prostate, colon, breast and cervical cancers, raise the question of whether HGSOC is amenable to similar screening strategies. Emerging insights into HGSOC’s disease progression suggest that early detection of low volume advanced stage, rather than large volume early stage HGSOC, may be a more clinically actionable goal of screening studies, since five-year relative survival rates for advanced stage cancers at diagnosis are significantly lower than for early stage cancers at diagnosis [2, 3, 5, 6, 8–10]. Moreover, HGSOC does not follow a clearly distinguishable pathologic continuum of neoplasia compared to, for instance, subtypes of breast, bowel or cervical cancers [6, 11], and detecting HGSOC in its non-specific early stage phase remains challenging [5, 10, 12]. These findings are especially relevant when evaluating the efficacy of transvaginal ultrasound (TVU)-based HGSOC detection, as TVU represents an integral part of all reported major ovarian cancer screening trials, despite its well-recognized limitations (e.g. bilateral disease, or multiple foci spread throughout the peritoneal cavity) [13]. TVU is accurate in detecting abnormalities in ovarian volume and morphology, but is less reliable in differentiating benign from malignant tumors [7, 8, 14–18]. As a result, whether HGSOC constitutes a valid target for ovarian cancer screening remains unanswered and highly contentious with respect to either general-risk or high genetic-risk women, such as germline *BRCA1* and *BRCA2* mutation carriers, or women with a significant family history of breast or ovarian cancer.

So far, evidence of a mortality benefit continues to elude HGSOC screening. Several studies have evaluated the efficacy of uni- or multi-modal TVU screening in general-risk populations and their impact on mortality benefit for several ovarian cancer histologies [14–16, 19, 20]. For example, in cohorts comprising of high genetic-risk women, multimodal conventional screening strategies failed to detect microscopic, early stage HGSOC tumor volumes [21–23]. In the Prostate, Lung, Colorectal and Ovarian Cancer Screening Trial (PLCO), simultaneous TVU and CA-125 screening in a general-risk population of women did not reduce overall mortality rates, compared to a group offered their usual medical care [14, 15]. This randomized controlled trial demonstrated that abnormal screening results led to unnecessary surgical procedures performed on false-positive women, a significant proportion of which subsequently experienced serious complications. More recently, data from the UK Collaborative Trial of Ovarian Cancer Screening (UKCTOCS), the largest ever such screening study performed to date, underscored the failure of unimodal TVU examinations to improve ovarian cancer detectability and overall survival rates [8, 24]. The study, comprising of 202,638 general risk women, demonstrated that multimodal screening including serial TVU and CA125 level testing yielded a 15% mortality reduction rate compared with a 0% no screening or 11% unimodal TVU-based screening cohort mortality reduction rate over 0–14 follow-up year. Lastly, the US Preventive Services Task Force (USPSTF) has recently reconfirmed their previous recommendation against ovarian cancer screening in asymptomatic women without known genetic mutations that increase their risk for ovarian cancer [25].

A wide variety of mathematical models on cancer and tumor growth exist, e.g. [26–29], however, few investigations have been concerned with any of the ovarian cancer subtypes. Current mathematical models address primary ovarian cancer tumor growth [30–32], sequencing of surgery and chemotherapy [33–35] or optimal characteristics of biomarkers [36], but there are limited data on ovarian cancer subtypes and the corresponding mathematical modeling of their growth kinetics. Although these models aim to reproduce HGSOC dynamics, HGSOC carcinogenesis is limited to being modeled as an exponential or logistic growth process [30, 31, 36]. Furthermore, the existing mathematical efforts conducted towards modeling ovarian carcinogenesis or estimating the efficacy of screening strategies do not properly across for the considerable inter-patient heterogeneity in malignancy initiation and progression. Lastly, none of the existing models includes a potential mechanism that correlates with the *in vitro* temporary cessation of tumor growth, or provides a quantitative assessment of the feasibility of frequent TVU monitoring with respect to HGSOC low-volume detectability and overall survival. It is precisely this absence of inferences from mathematical modeling regarding HGSOC progression that motivated this study of TVU-based detection strategies.

Herein, we propose a novel *in silico* mathematical model that provides a quantitative explanation behind the reported failure of TVU to improve HGSOC low-volume detectability and overall survival rates. We develop a mathematical assessment of the efficacy of a unimodal TVU monitoring regimen as a strategy aimed at detecting low-volume HGSOCs in cancer-positive cases, defined as cases in whom the inception of the first HGSOC malignant cell has already occurred. Our model captures the dynamic, temporal evolution of HGSOC growth and progression, and provides quantitative estimates of otherwise unknown clinical parameters such as the duration of HGSOC’s pre-diagnosis stage and the screening window of opportunity interval length.

## Methods

We develop an *in silico* mathematical framework modeling incipient HGSOC growth kinetics in an untreated scenario, subject to stochastic heterogeneous fluctuations. Herein, we refer to an untreated HGSOC as a radiographically detected, clinically asymptomatic, treatment-free malignancy in which no surgery and/or other systemic therapies has yet been performed/administered. Inspired by a stochastic numerical model of breast cancer growth [37], we follow a similar approach to model HGSOC natural history and progression until clinical TVU detectability. The key feature of this model incorporated in the present work involves modeling HGSOC progression as Gompertzian growth kinetics that is further characterized by infrequent, rate-limiting stochastic changes in the growth parameters.

### HGSOC growth rate estimation

**Fig 1:**
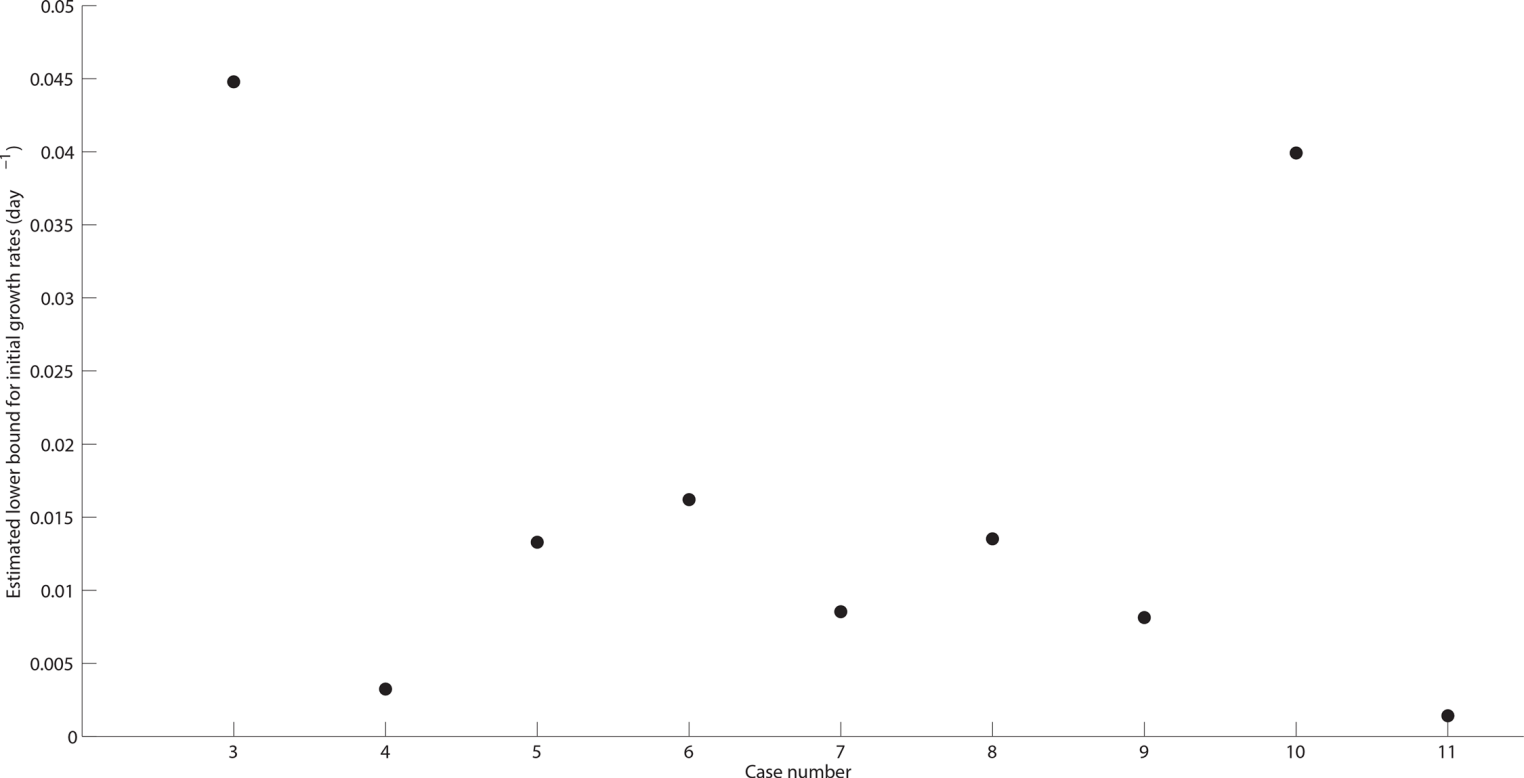
Estimated lower bounds for HGSOC initial tumor growth rates–derived from reported TVU findings of 9 previously undetected HGSOC primary tumor sizes [40]. Reported bi-dimensional measurements of 9 incidental, previously undetected HGSOC tumor sizes (length, width) were converted into a weighted, one dimensional measurement (i.e. spherical radius): weighted radius $\text{r}=\;\sqrt{a\cdot b}$, where *a* and *b* are the radii of the minor and major axes of an ellipsoid, respectively. *r* represents the weighted radius of the reported HGSOC tumor sizes (as derived from Table 1 in [40]). To compute HGSOC initial tumor volumes, we assume tumors to be spherical and compute their volume according to the formula $\frac{4\pi }{3}\cdot r^{3}\;(cm^{3})$. We assume normal ovarian volumes were 20 cm^3^ for the pre- and 10 cm^3^ for the postmenopausal women reported in Table 1 of [37]. We estimate the lower bounds for the initial HGSOC growth rates according to the following formula: $\text{initial growth rate}=\frac{\text{ln}(\text{tumor volume at diagnosis})\;-\text{ln}(\text{normal ovarian volume})}{\text{T}}$, where *T* represents the number of days between the timing of the previous, non-suspicious TVU examination and the malignancy diagnosis, converted from the number of months corresponding to each patient case as reported in [40] (median HGSOC initial growth rate = 0.0133 day^-1^, range = 0.0014–0.0448). Each point plotted (case number, estimated lower bound for initial growth rate) corresponds to case number (3–11) reported in Table 1 of [40].

To estimate a lower bound for the initial HGSOC growth rates, we identified the existing TVU-based screening study with the largest cohort of ovarian cancer patients [17]. In this study, data concerning ovarian volumes were obtained from 13,963 patients who were undergoing annual TVU examinations from 1 to 11 years. We define abnormal ovarian enlargement as two standard deviations above normal ovarian volume in pre- and postmenopausal women, see [38]. Based on 58,673 ovarian volume observations, the upper limit for normal ovarian volume therein was found to be 20 cm^3^ for pre- and 10 cm^3^ for postmenopausal women [38]. Menopause is defined as occurring 12 months after a woman’s last menstrual cycle and confirmed by follicle stimulating hormone levels > 40 IU/L [39]. We subsequently assume that any HGSOC tumor volume larger than the difference between the two pre-defined thresholds (i.e. 10 cm^3^) would represent a suspicious TVU finding, and subsequently be diagnosed as a radiographically detectable HGSOC case. The data points illustrated in Fig 1 represent estimated lower bounds for the initial HGSOC rates used to initialize our model. They correspond to 9 reported HGSOC clinical findings based on TVU examinations of adnexal ovarian regions available 12 months or fewer prior to the preoperative diagnosis time of the malignancy [40]. The reported cases showed no apparent ovarian volume abnormalities 2 to 12 months prior to TVU diagnosis. We note that, to the best of our knowledge, these findings represent the only available temporal data on the progression of previously occult, radiographically detected HGSOCs.

**Table 1:** Definitions used throughout the model.

Term	Definition
Occult growth curve	*in silico* HGSOC growth curve that never reaches TVU detectability.
Succumbed growth curve	*in silico* HGSOC growth curve that reaches both a TVU detectable and life-threatening tumor volume, see [41], in between consecutive TVU monitoring events.
Cancer-positive growth curve	*in silico* HGSOC growth curve in which the inception of the first HGSOC malignant cell has already occurred.
Untreated growth curve	*in silico* HGSOC growth curve described as a radiographically detected, treatment-free malignancy in which no surgery/therapy has yet been performed/administered.

HGSOC-growth curve time is measured from the inception of the first malignant cell until the time needed to reach the baseline TVU detection threshold, or until the baseline life-threatening tumor volume is reached. Herein, we assume that the minimum, baseline TVU detectability threshold for a cancer-positive case is 10 cm^3^ (equivalent to 10^10^ cells, or to a 2.673 cm spherical HGSOC tumor diameter. Similarly, we follow the definition of the life-threatening untreated HGSOC tumor volume to be 10^3^ cm^3^ (equivalent to 10^12^ cells, or to a 12.407 cm spherical tumor diameter), as previously published [41]. The two thresholds can be adjusted if more sensitive diagnostic techniques are developed, or if different life-threatening untreated HGSOC tumor volume values are used. We assume the cell number-to-volume conversion to be 1 cm^3^ = 1 cc = 10^9^ HGSOC cells [42]. The baseline thresholds were chosen to estimate conservative lower bounds for the time of TVU diagnosis and time of reaching the life-threatening tumor volume distributions. Herein, we define the *window of opportunity* interval as the difference between the two thresholds based on the growth curves that reach both endpoints.

### Modeling equations

We use the incipient HGSOC growth kinetics model to study the timing of HGSOC initiation relative to reaching TVU detectability and the life-threatening untreated tumor volume sizes, as defined above, and its subsequent implications on TVU monitoring protocols. We choose to use the terminology ‘TVU monitoring’ in lieu of ‘TVU screening’, as the latter would be a more appropriate term for a detection strategy focused on a cohort of cancer-negative, general or high-risk otherwise asymptomatic healthy women [18], as opposed to a pre-selected, biased *in silico* cancer-positive cohort, for which the former term is more appropriate.

A main study end point for this model was HGSOC-specific mortality, specifically the number of *in silico* HGSOC growth curves that would be missed even under frequent TVU monitoring. To this end, we developed a mathematical framework modeling incipient untreated HGSOC volume growth in order to satisfy two purposes: one, to simulate the natural history of the malignancy, and two, to quantify the relationship between TVU monitoring frequency and detection time of a non-life-threatening HGSOC volume.

To obtain a temporal estimate of the effective growth behavior of a simulated HGSOC growth curve, we let N(t) be the total HGSOC tumor volume, i.e. the number of HGSOC cells located in the primary tumor site (e.g. one of the ovaries, or the fallopian tubes), at time t. N_0_ represents the initial, pre-diagnosis HGSOC cell count, set as 1 for computational convenience, and time t is measured since the inception of the first malignant cell. If we let k_growth_ represent the initial HGSOC growth rate constant and k_decay_ describe the growth saturation rate, where both parameters have the dimension of inverse time (e.g. in our case, day^-1^), the Gompertz function modeling tumor growth can be expressed as

$$\text{N}\left(\text{t}\right)={\text{N}}_{0}{\text{e}}^{\frac{{\text{k}}_{\text{growth}}}{{\text{k}}_{\text{decay}}}\left(1-{\text{e}}^{-{\text{k}}_{\text{decay}}\;\text{t}}\;\right)},\;\text{N}\left(0\right)=1.$$ (1)

The normalized N (t) thus satisfies the following differential equation:

$$\frac{\text{dN}}{\text{dt}}=\text{N}\left(\text{t}\right)\cdot \left[{\text{k}}_{\text{growth}}-{\text{k}}_{\text{decay}}\cdot \text{ln}\;\text{N}\left(\text{t}\right)\right].$$ (2)

The carrying capacity N(∞) = N_∞_ is assumed to be finite and nonzero. It follows that k_decay_ > 0, and that $\text{N}\left(\infty \right)={\text{e}}^{\frac{{\text{k}}_{\text{growth}}}{{\text{k}}_{\text{decay}}}}>1$. To find the inflection point of N(t), that is N_i_(t_i_), we require

$$\frac{{\text{d}}^{2}\text{N}}{\text{d}{\text{t}}^{2}}=\;\left({\text{k}}_{\text{growth}}-{\text{k}}_{\text{decay}}\cdot \text{ln}\;\text{N}\left(\text{t}\right)-{\text{k}}_{\text{decay}}\right)\cdot \;\frac{\text{dN}}{\text{dt}}=0,$$

i.e., the derivative of the change in HGSOC growth rate is set as 0. Since N(t) > 0 for finite t, then

$$\text{ln}\;{\text{N}}_{\text{i}}=\frac{{\text{k}}_{\text{growth}}}{{\text{k}}_{\text{decay}}}-\;1,{\text{t}}_{\text{i}}=\frac{1}{{\text{k}}_{\text{decay}}}\cdot \text{ln}\frac{{\text{k}}_{\text{growth}}}{{\text{k}}_{\text{decay}}}.$$ (3)

$$\text{It thus follows that}\;{\text{N}}_{\text{i}}={\text{e}}^{\frac{{\text{k}}_{\text{growth}}}{{\text{k}}_{\text{decay}}}-1},{\text{N}}_{\infty }=\text{e}\;{\text{N}}_{\text{i}}.$$ (4)

In this case, the tumor cell burden can outgrow its size at the inflection point by a factor of e. The inflection point represents a turning point in the dynamics when the observed growth trend starts decelerating. Nonetheless, while the Gompertz equation describes a density-dependent growth rate, it does not account for any stochastic irregularities, e.g. stepwise growth patterns, also see [37]; such temporary Gompertzian plateaus (i.e. cessation in tumor growth) may be correlated, as reported *in vitro*, with tumor dormancy in ovarian cancer spheroids [43, 44], human ovarian cancer cell lines [45, 46], or *in vivo* with tumor xenografts implanted in mice [45], and may be associated with dormancy in untreated, undetectable HGSOC. A constant growth rate might not be feasible to model progression. To this end, by incorporating rare but relatively large jumps in the growth saturation rate k_decay_, we assume that HGSOC growth slows down due to adverse environmental conditions (e.g. reactive *O*_2_ presence, nutrient depletion). The irregular tumor growth kinetics illustrated in our model accounts for the observed heterogeneity in the progression of clinical HGSOCs [47] and highlights the differential HGSOC natural histories that lead to identical clinical outcomes or presentations (e.g. see case numbers (4) and (11) reported in Table 1 of [40]). The tumor growth kinetics represented herein could thus be phenomenologically valid both *in vivo* and *in vitro*.

### Modeling assumptions

We assume the inception of the first HGSOC malignant cell occurs sometimes during premenopausal years, and thus we increment time in intervals of 28 days (the average length of a menstrual cycle [48]) for a total number of 460 menstrual cycles, the average cumulative length of a lifetime menstrual cycle. We set the initial k_decay_ to be initial $\frac{{\text{k}}_{\text{growth}}}{2}$. Varying this initial parameter would not yield substantially different median or range values for the estimated cdf’s. We then implement the changes in the initial growth saturation rate, k_decay_, in a two-step manner. First, we generate a number α ∼ ln N(10^−2^, 25 ⋅ 10^−2^), that is log-normally distributed with mean = 10^−2^, variance = 25 x 10^−2^, and range = 0.0094 − 0.150 (0.01 mean probability of change in each 28-day period, or 26% mean probability of change in 2 years). Herein, α refers to the probability of random change in k_decay_. In order to implement conservative estimates for the clinically occult random variable α, we choose an asymmetric, right-skewed probability distribution function. Second, we check whether α is less than a randomly generated number between 0 and 1. If that is the case, we then generate a second random number between 0 and 1 and compute the updated k_decay_ as

$${\text{k}}_{\text{decay}}=\frac{\text{previous}\;{\text{k}}_{\text{decay}}}{1+\text{random number}}.$$ (5)

We then allow the number of HGSOC cells, N(t), to follow the Gompertzian growth law until the probability of a random change in k_decay_ occurs again, which leads to another update.

Given a fixed carrying capacity, varying either k_growth_ or k_decay_ makes little qualitative difference from a mathematical perspective; we can thus infer that modifying either parameter yields similar qualitative effects. From a molecular perspective, we chose to focus on changes in the initial HGSOC growth saturation rate, k_decay_, as these infrequent, rate-limiting changes could be associated in part with the several (epi)genetic alterations in tumor suppressor genes and/or changes in genes involved in DNA damage repair pathways. Reducing the growth saturation rate, k_decay_, of the HGSOC tumor cell burden the program increases the current HGSOC carrying capacity, $\text{N}\left(\infty \right)={\text{e}}^{\frac{{\text{k}}_{\text{growth}}}{{\text{k}}_{\text{decay}}}}$, in a stochastic fashion. Changes are globally implemented, meaning that once a stochastic jump in *k*_*decay*_ occurs, cells proliferate according to the newly updated Gompertz-type growth law. Simulation time continues until the untreated HGSOC life-threatening tumor volume threshold is reached (e.g. corresponding to 10^12^ HGSOC cells), or until 38.5 years since the inception of the first HGSOC cell have elapsed. If the respective HGSOC growth curve reaches TVU detectability, we compute the time since the inception of the first HGSOC cell until clinical detection is reached. Similarly, we compute the time until clinical life-threatening HGSOC tumor volume is reached if the respective HGSOC growth curve reaches that stage. For an individual growth curve, the initial *k*_*growth*_ is uniformly sampled from the values illustrated in Fig 1. Calculations are performed for *n = 1000* simulated growth curves. A flowchart of the computational model is shown in Fig A in S1 File. The definitions and assumptions used throughout the implementation of the HGSOC carcinogenesis, growth and progression model are provided in Tables 1 and 2, respectively.

**Table 2:** Assumptions used throughout the model.

Assumption	
1	Patients under 50 years of age are assumed premenopausal; conversely, patients over 50 are assumed postmenopausal. Herein, menopause is defined as occurring 12 months after a patient’s last menstrual cycle, and confirmed by follicle stimulating hormone levels > 40 IU/L [39, 48].
2	The average length of a woman’s menstrual cycle is 28 days [48]; the lifetime cumulative number of menstrual cycle a woman with two full-term pregnancies experiences is 460, equivalent to a cumulative lifetime period of 38.5 years.
3	Ovarian volumes >20 cm^3^ in premenopausal and >10 cm^3^ in postmenopausal women are defined as abnormal [38], and are assumed in this model to be indicative of positive disease.
4	HGSOC cells are assumed to exhibit indefinite proliferative potential [49]; for simplicity, we assume no explicit spatial constraints.
5	The initial TVU monitoring event for each simulated HGSOC growth curve occurs at a time point randomly distributed between 0 and 5 years since the inception of the first HGSOC cell; times needed to reach TVU detectability, the life-threatening untreated volume and window of opportunity interval length are recorded from subsequent monitoring events until the end of simulation time.
6	The TVU monitoring algorithm has 100% specificity and 100% positive predictive value.
7	All HGSOC growth curves are compliant with the TVU monitoring protocol (i.e. perfect detection times are reported).
8	Contamination at TVU monitoring is 0 (i.e. no incidental TVU examinations are performed between consecutive monitoring events).
9	All simulated HGSOC growth curves refer to untreated cases. This enables us to provide an estimate of the window of opportunity interval length in the absence of any treatment.
10	If left untreated, all HGSOCs detected by the TVU monitoring events can potentially lead to a life-threatening tumor volume load; HGSOC growth curves defined as occult are assumed to remain clinically dormant and non-life-threatening for the entire duration of simulation timeframe.

## Results

### Model simulation of HGSOC *in silico* growth curves

**Fig 2:**
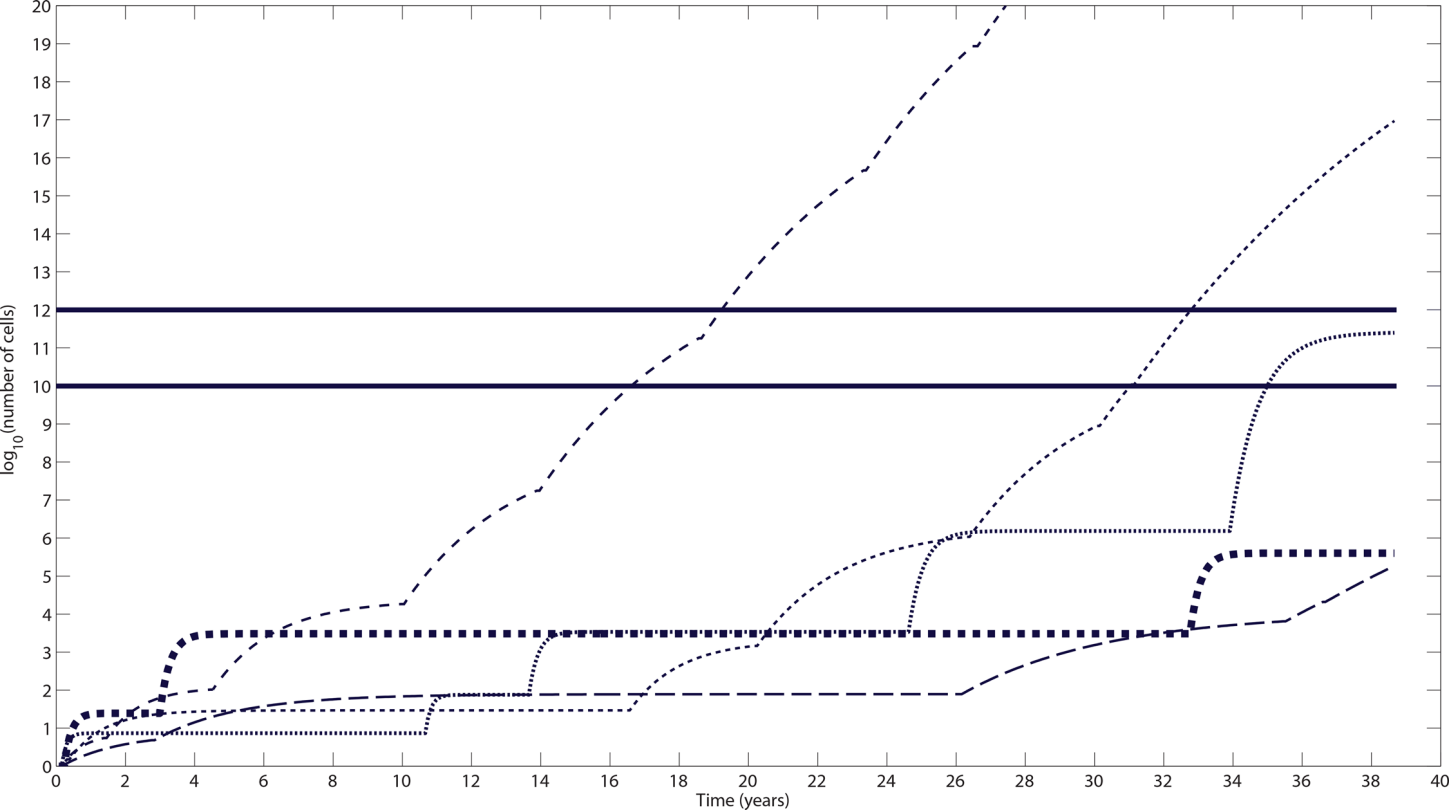
Representative simulation of the *in silico* HGSOC growth and progression model generating five HGSOC growth curves. This sample simulation of the HGSOC progression model illustrating five representative growth and progression curves is generated using the same baseline parameter set outlined in Tables A-B in S1. Each of the representative growth curves is an independent realization of the HGSOC stochastic growth and progression model, initialized with the same parameter value set. Values the baseline TVU detectability and life-threatening untreated tumor threshold are as reported previously. In this representative simulation, two curves reach the detection threshold (lower solid line) in 16.4 and 31.2 years, respectively, and life-threatening tumor volume threshold (upper solid line) in 19.3 and 32.6 years, respectively. The calculated window of opportunity interval length is thus 2.9 and 1.4 years, respectively. One curve reaches only the detection threshold, in 35.0 years, and two curves remain below both thresholds. Time is measured since the inception of the first HGSOC cell. The curves are sorted from left to right. Note that the probability that a random change in k_decay_ occurs is independent of whether current carrying capacity is reached or not.

Based on the HGSOC clinical findings reported in [40] upon TVU examinations 12 months or fewer prior to the diagnosis time of the malignancy, we computed a median HSGOC initial growth rate of k_growth_ = 0.0133 day^-1^ (range = 0.0014–0.0448). The data points illustrated in Fig 1 represent estimated lower bounds for the initial HGSOC rates used to initialize our model. Five representative growth curves generated by the HGSOC model in our simulated cancer-positive cohort are illustrated in Fig 2. The same baseline parameter set and cell-number-to-volume and tumor diameters conversions were used (Tables A–B in S1 File). By incorporating rare but relatively large jumps in the growth saturation rate k_decay_, we illustrate how a HGSOC volume grows in stepwise patterns and may not increase for relatively large amount of time (Fig 2), as opposed to exhibiting a constant doubling time. This approach also enables us to generate a distribution of heterogeneous pre-clinical HGSOC natural histories in an *in silico* cancer-positive cohort. Statistics generated from one representative simulation of the HGSOC growth and progression model using *n = 1000* HGSOC growth curves are reported in Table B in S1 File. Therein, the generated data illustrate the time needed to reach the baseline TVU-detectable HGSOC volume of 10 cm^3^, the baseline life-threatening tumor volume of 10^3^ cm^3^, and the window of opportunity interval length. The number of HGSOC growth curves that never reach the TVU baseline detectability threshold (occult), or the life-threatening threshold (succumbed) are also reported therein. Subsequent results reported below are based on the same computation that yielded the data generated in Table B in S1 File.

### Number of HGSOC carcinogenetic events leading to HGSOC growth and progression

**Fig 3:**
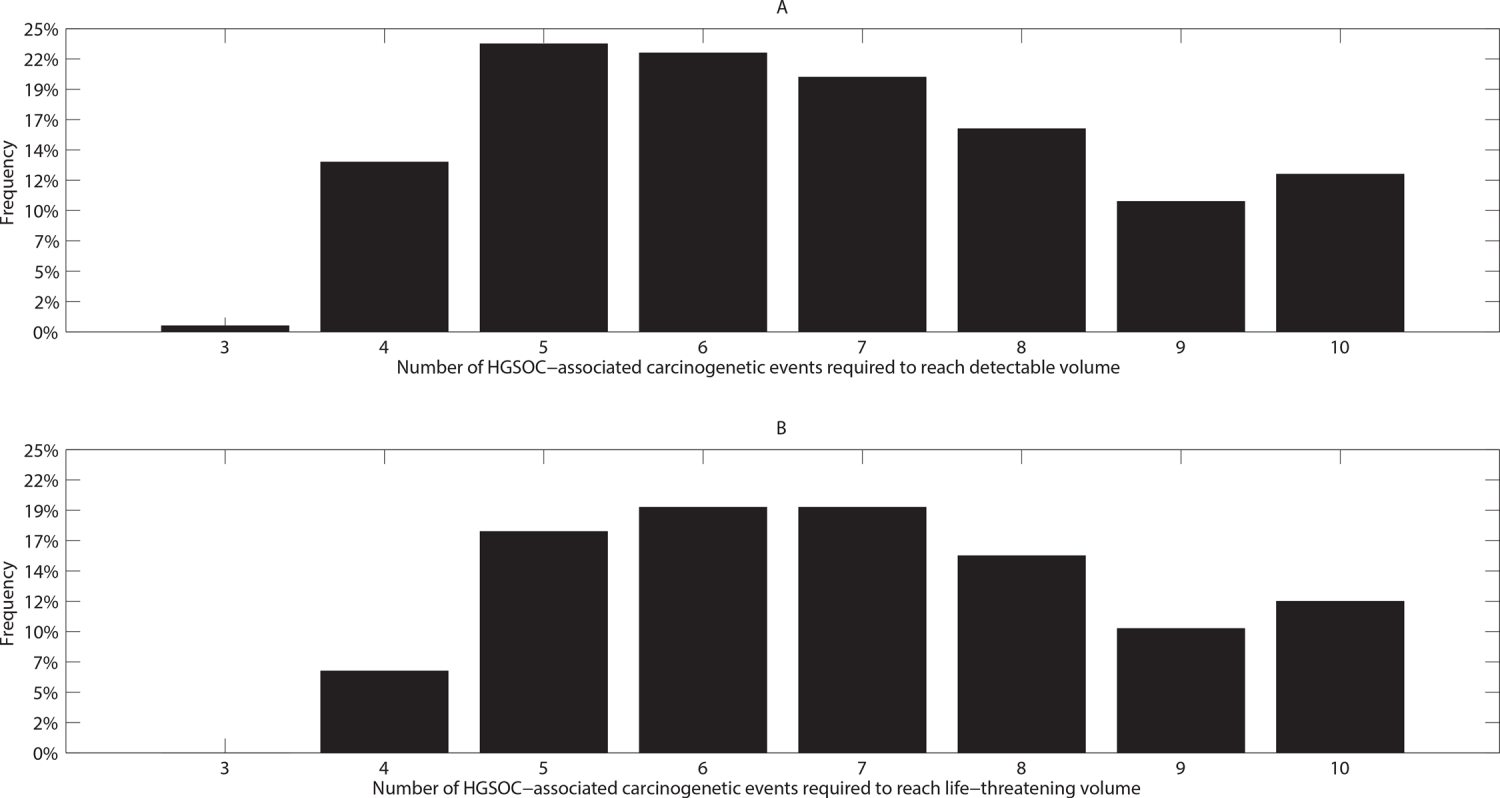
Number of HGSOC carcinogenetic events leading to HGSOC growth and progression. In one representative simulation of the model generating 1000 cancer-positive initially clinically occult HGSOC growth curves, (A) 491 sample HGSOC growth curves progress to reach the detectability threshold and (B) 418 reach the life-threatening volume threshold. (A) We record the frequency of rate-limiting events associated with changes in the initial growth saturation rate, k_decay_ out of the *n = 491* growth curves. For this representative simulation, the mode and median number of such events are 5, and 7 respectively. (B) We record the number of rate-limiting events associated with changes in the initial growth saturation rate, k_decay_, out of *n = 418* growth curves. For this representative simulation, the mode and median of the number of such events are 7, and 7 respectively. A maximum number of 10 events associated with changes in the initial growth saturation rate, k_decay_, is recorded in both panels.

Computational results indicate that for the 491 sample HGSOC growth curves that reach the baseline TVU detection threshold, the number of infrequent, rate-limiting events associated with changes in the initial growth saturation rate, k_decay_ is around 7 (median = 7, mode = 6, range = 3–10, Fig 3A). Interestingly, for this representative simulation, the mode number of required events was 5, and the reported maximum of such events was 10. Note also the substantial heterogeneity in the number of events required to lead to a TVU-detectable HGSOC tumor volume. Similarly, for the 418 growth curves that reach the baseline life-threatening tumor volume threshold, the number of rate-limiting events associated with changes in the initial growth saturation rate, k_decay_, is around 7 (median = mode = 7, range = 4–10, Fig 3B), and the reported maximum of such events was 10. Note again the substantial heterogeneity in the number of events required to lead to a life-threatening, untreated HGSOC tumor volume since the inception of the first malignant cell. One or two *extra* events are required in order for a detectable HGSOC tumor volume to become life-threatening.

### Estimating the window of opportunity interval length

**Fig 4:**
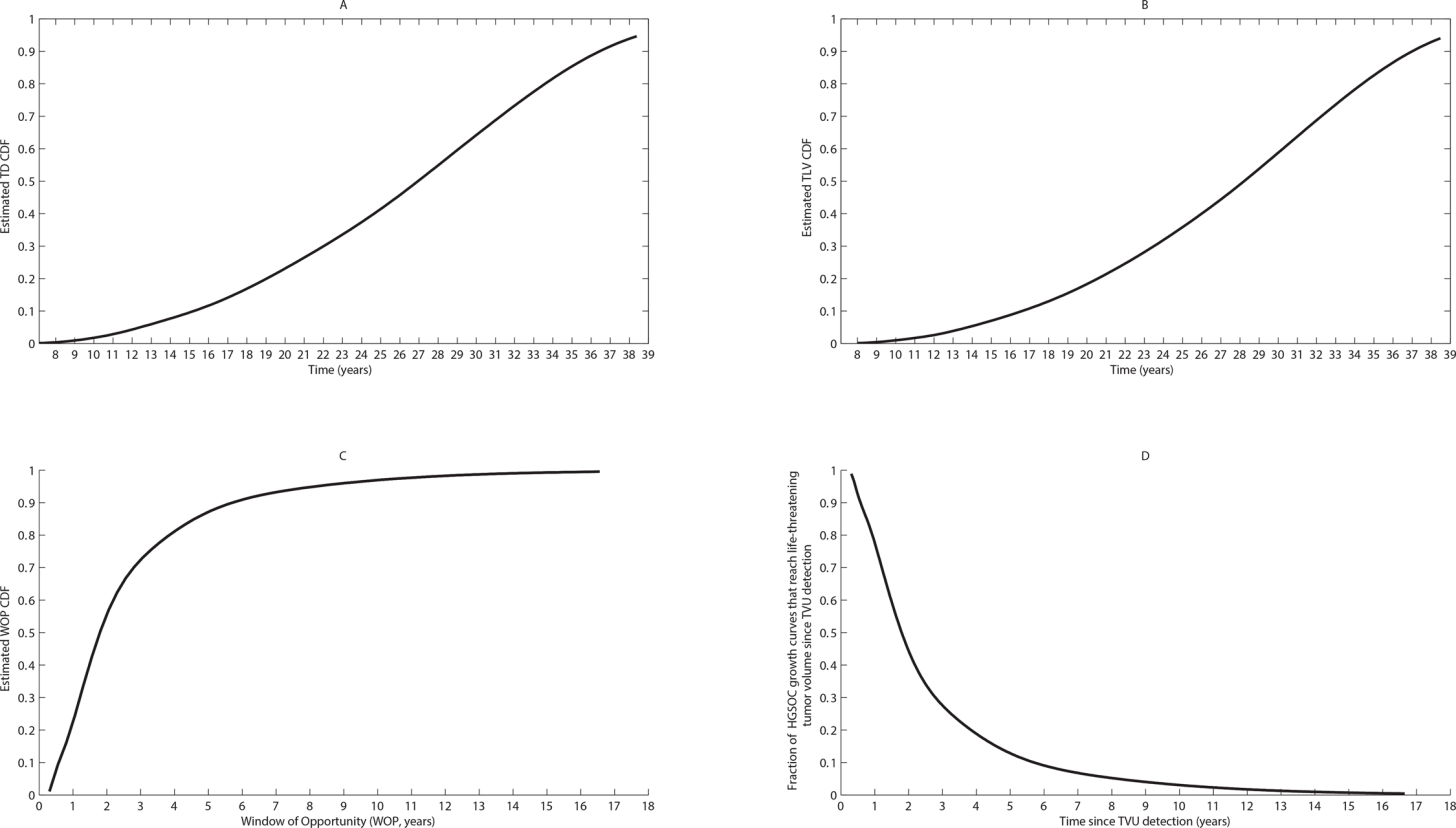
**Empirical cumulative distribution functions for (A) time until baseline TVU detection threshold is reached (TD); (B) time until life-threatening tumor volume is reached (TLV); (C) the window of opportunity interval length; (D) the fraction of radiographically detected, treatment-free HGSOC cases that progressively reach the life-threatening threshold starting from the baseline detection threshold time.** The progression of *n = 1000* HGSOC growth curves is simulated in order to determine typical empirical cumulative distribution functions for (A) time until baseline TVU detection threshold is reached (median = 26.7 years, range = 4.52–38.5); in this sample simulation, a total of 498 growth curves reach this threshold; (B) time until life-threatening tumor volume is reached (median = 27.65 years, range = 7.28–38.6). In this sample simulation, a total of 418 growth curves reach this threshold; (C) window of opportunity interval length (median = 1.76 years, range = 0.3–14). In this sample simulation, a total of 418 growth curves reach both the baseline detection and life-threatening volume thresholds, and are thus included in the window of opportunity interval length calculation. Statistics generated from one sample simulation of the HGSOC growth and progression model illustrating the time needed to reach the baseline TVU detection threshold, the baseline life-threatening tumor volume (TLV), the window of opportunity interval length (WOP), and the number of HGSOC growth curves that never reach TVU baseline detectability (occult), or the life-threatening threshold (regressed) volumes, respectively, during one sample simulation are given in Table C in S1 File.

To produce estimates of the duration of HGSOC’s pre- and post-diagnosis phases, we report the generated value ranges (median, range), with median values of the times needed to reach baseline TVU detection threshold, baseline life-threatening tumor volume, and the window of opportunity interval length; we chose to report median values as the median was a more robust statistic compared to the mean throughout all sample model simulations, and thus constitutes a more accurate descriptor of the aggregate cancer-positive HGSOC population dynamics. The model-generated empirical cumulative distribution functions (cdf’s) for reaching the baseline detection threshold, the baseline life-threatening tumor volume and the window of opportunity length interval are reported in Fig 4A–4D. For this representative simulation, a total of 498 growth curves reach the baseline TVU detection threshold (median = 26.7 years, range = 4.52–38.5), a total of 418 growth curves reach the baseline life-threatening tumor volume threshold (median = 27.65 years, range = 7.28–38.6), and a total of 418 growth curves reach both thresholds, and are thus included in the window of opportunity interval length computation and cdf estimation (median = 1.76 years, range = 0.3–14, Fig 4C). As an alternative to Fig 4C, we illustrate in Fig 4D the fraction of radiographically detected, treatment-free HGSOC growth curves that progress to the life-threatening volume threshold is illustrated. Increasing the number of simulated HGSOC growth curves (*n > 1000)* does not yield substantially different median or range values for the estimated cdf’s.

### Assessing the feasibility of multiple frequency TVU monitoring protocols

**Fig 5:**
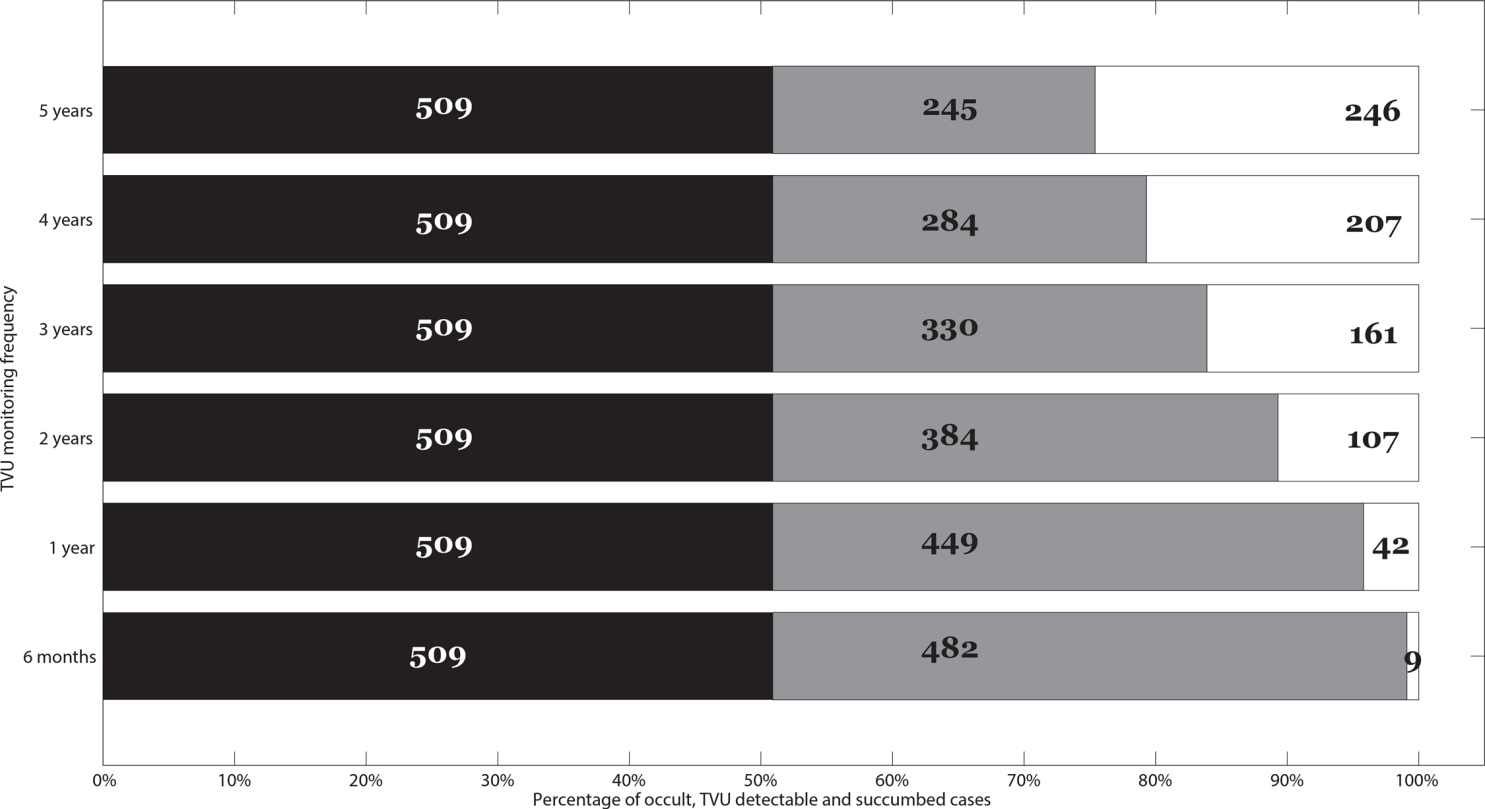
Feasibility of HGSOC unimodal TVU monitoring across multiple monitoring frequencies. We report the relative proportions of HGSOC curves that remain occult (first, black horizontal column), that are detectable in the first or subsequent TVU monitoring events (second, grey horizontal column), and lastly, that are succumbed (third, white horizontal column) out of *n = 1000* initial HGSOC growth curves. The proportions reported vary for different TVU monitoring frequencies, i.e. every 5, 4, 3, 2 years, every 1 year (annually) or every 6 months (semiannually). Importantly, the last horizontal column represents the percentage of HGSOC growth curves that would be missed even under frequent TVU monitoring.

Fig 5 illustrates the relative proportions of HGSOC curves that remain occult (first, black horizontal column), that are detectable in the first or subsequent TVU monitoring events (second, grey horizontal column), and lastly, that are succumbed (third, white horizontal column) out of *n = 1000* simulated HGSOC growth curves. Semiannual monitoring HGSOC progression via TVU performs the best (0.9% of total HGSOC curves succumb [see Table 1 for definitions]) despite the frequent TVU monitoring, compared to a 4.2% succumb rate when monitored annually, or 10.7% when monitored biannually. It is also worth noting the relatively large proportion of HGSOC curves that remain occult (50.9% of the total *n = 1000* growth curves in this representative simulation, Fig 5). This representative simulation was performed using the baseline parameters outlined in Tables A-B in S1 File.

### TVU Sensitivity Analysis

**Fig 6:**
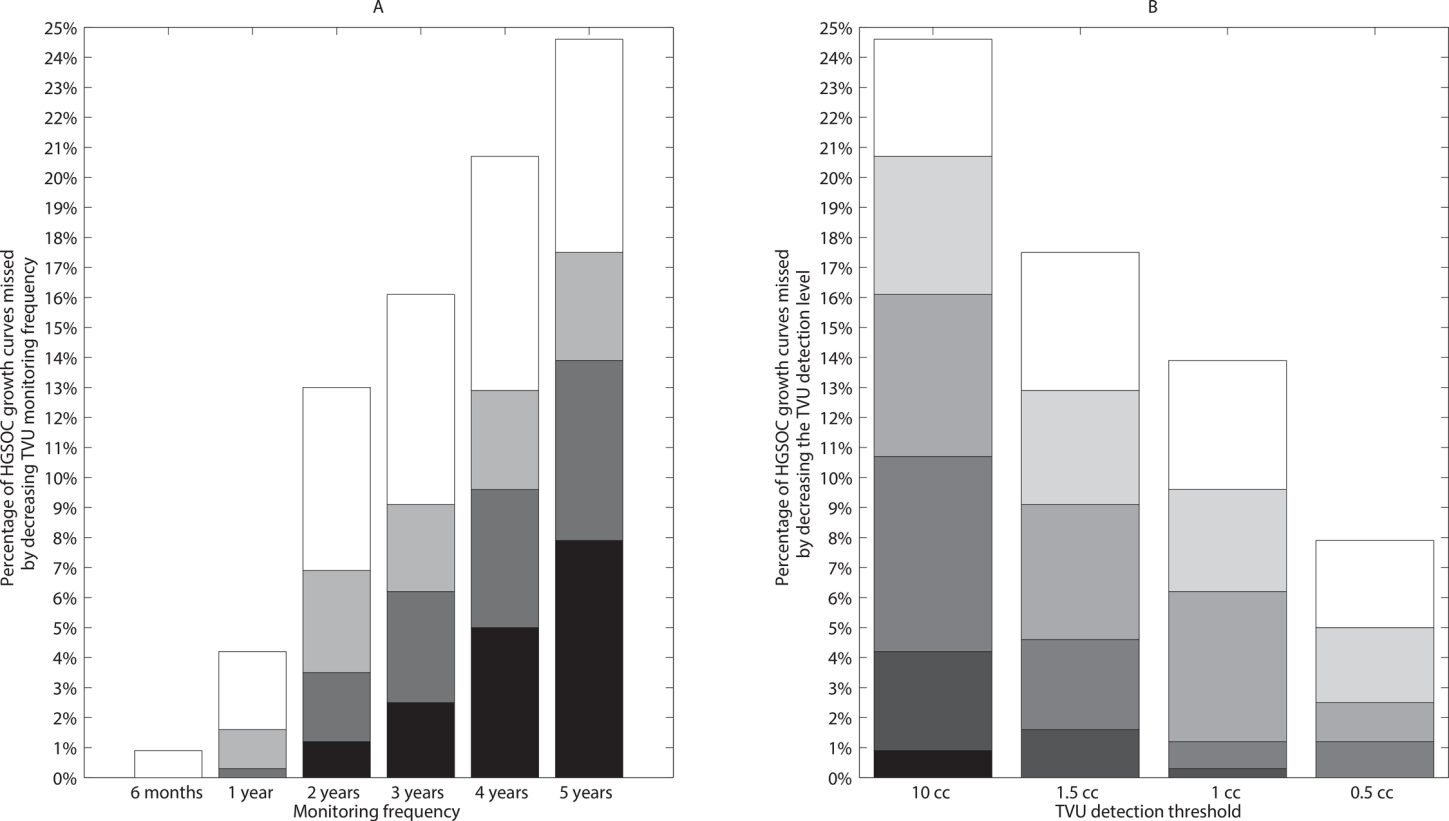
**Percentage of HGSOC growth curves that are not detected by TVU monitoring for (A) varying monitoring frequencies and (B) varying TVU detection thresholds.** (A) Fewer HGSOC growth curves reach the succumbed status with more frequent TVU monitoring events (x-axis) and more sensitive TVU detection thresholds (0.5 cm^3^, black vertical columns; 1 cm^3^, dark grey vertical columns; 1.5 cm^3^, light grey vertical columns; 10 cm^3^, white vertical columns). In this panel, vertical columns indicate the percentage of additional HGSOC growth curves that are missed by decreasing TVU detection thresholds relative to the baseline TVU detection threshold set at 10cm^3^, out of a total of 1000 simulated HGSOC growth curves. (B) Fewer HGSOC growth curves reach the succumbed status with more sensitive TVU detection thresholds (x-axis) and more frequent TVU monitoring events ranging from six months to five years. In this panel, vertical columns indicate the percentage of additional HGSOC growth curves that are missed by decreasing the frequency of TVU monitoring events relative to a baseline 6-month frequency, out of a total of 1000 simulated HGSOC growth curves.

We conducted a sensitivity analysis with respect to the TVU detection thresholds, set at 0.5, 1 or 1.5 cm^3^, to determine whether the percentages reported above would drastically vary. We demonstrate that the percentage of HGSOC growth curves that reach the updated baseline detection and life-threatening tumor volume thresholds in between the same monitoring events increases with less frequent TVU monitoring events (Fig 6A) and decreases with more sensitive TVU detection thresholds (Fig 6B). Our findings confirm that more sensitive TVU detection thresholds and more frequent TVU monitoring improve diagnostic accuracy (decreasing the number of succumbed HGSOC growth curves). These plots were generated from one representative simulation using a total number of *n = 1000* simulated growth curves and performed using the same baseline parameter set and cell-number-to-volume and tumor diameters conversions as reported in Tables A-B in S1 File. The data used to produce Fig 6 is given in Table D in S1 File.

## Discussion

HGSOC constitutes an attractive target for early detection strategies if detected before reaching large volume advanced stage, when overall survival rates are grim [50]. The validation of any HGSOC tumor volume clinical detection strategy is thus whether frequent screening is capable of lowering mortality rates. However, numerous transvaginal ultrasound (TVU) detection-based population studies aimed at detecting low-volume ovarian cancer have not yielded reduced mortality rates and thus challenge the effectiveness of TVU as a HGSOC monitoring strategy aimed at improving overall survival rates [7, 8, 14–17, 20–23, 40, 51]. A quantitative invalidation of TVU as an effective HGSOC screening strategy is a necessary next step. Our mathematical modeling approach proposes a quantitative explanation for the reported failure of TVU to improve HGSOC low-volume detectability and overall survival.

We develop a novel *in silico* mathematical assessment of the efficacy of a unimodal TVU monitoring regimen as a strategy aimed at detecting low-volume HGSOCs in cancer-positive cases; our model captures the dynamic, temporal evolution of HGSOC progression, and is characterized by several rare rate-limiting events, which can be associated in part with (epi)genetic alterations in tumor suppressor genes and DNA damage repair pathways. We chose to focus on an unimodal, ultrasound-based HGSOC detection method (i.e. TVU), rather than on blood biomarker levels (i.e. CA-125 or HE4 levels), pelvic examinations or simultaneous TVU and CA-125 detection. Despite its well-recognized detection limitations in detecting localized or distant metastatic burden, TVU examinations are routinely performed when assessing ovarian volume, while the latter are either not recommended as low HGSOC volume detection unimodal prognostic markers [7, 15, 25] or have not been shown to confer a mortality benefit [15, 16]. Our results suggest that multiple frequency TVU monitoring across various detection sensitivities does not significantly improve detection accuracy of HGSOCs in an *in silico* cancer-positive population. Specifically, despite the fact that semiannual monitoring HGSOC progression via TVU performs, as expected, the best compared with annual or biannual monitoring (0.9% succumbed cases versus 4.2% and 10.7%, respectively), a nonzero percentage of succumbed cases is reported in all subsequent simulations of the HGSOC growth and progression model. Given that our TVU monitoring algorithm is assumed to have 100% specificity and 100% positive predictive value, the actual percentage of such succumbed HGSOC cases might be substantially higher. This invalidates the use of TVU as an effective HGSOC screening strategy aimed at lowering mortality rates in general-risk or high genetic-risk women. Our mathematical model thus represents a novel attempt to explain why multiple, large-scale TVU-based HGSOC detection screening studies have not proven significant mortality benefits, and focuses on a malignancy that has received very little attention by the mathematical oncology community.

We find that the median time until baseline TVU detection from the inception of the first HGSOC cell is 26.7 years. Given that an average patient’s age at diagnosis of ovarian cancer is 55–65 years [1], our findings suggest that the first HGSOC cell may appear on average around 28–38 years of age, during a patient’s premenopausal period. This may be due to a number of factors, including reproductive history, oral contraceptive use and family history of breast or ovarian cancer [52, 53]. Furthermore, simulation results suggest that once a HGSOC tumor volume becomes clinically detectable, it takes an additional median number of 1.7 years to reach the baseline life-threatening tumor volume threshold; this implies that for a radiographically detected, treatment-free malignancy in which no surgery and/or systemic therapies have yet been performed/administered, the patient would succumb to the disease relatively quickly after initial diagnosis. Since 90% of the diagnosed HGSOC patients do not have abnormal clinical findings based on TVU performed 12 months or more prior to HGSOC diagnosis [40], the reported median window of opportunity interval length (1.76 years) reflects a bias towards the more aggressive and fast-growing HGSOCs. This is a key prediction of our model, provided by computer simulations in the absence of clinical/experimental estimates of the period of time needed to reach the life-threatening tumor volume threshold or window of opportunity interval length. This does not, however, translate into reduced mortality levels in an *in silico* cohort across multiple TVU monitoring frequencies or detection sensitivities. Our findings suggest that even a semiannual, unimodal TVU monitoring protocol is expected to miss detectable HGSOCs. We also find that circa 50% of the simulated HGSOC growth curves never reach the baseline detectability threshold, and that on average, 5–7 rate-limiting events are associated with reaching HGSOC detectability and life-threatening untreated HGSOC volumes respectively.

The predictions obtained with our HGSOC model are consistent with other published cancer progression chronologies reported for colorectal [54] or pancreatic cancers [54, 55]. *Yachida et al*. analyzed genomic sequencing data of metastatic tumors from 7 patients with metastatic pancreatic cancer and calculated that the first parental (non-metastatic) founder cancer cell may require 6.8 years to generate sub-clones with metastatic potential [55]. These sub-clones could give rise to distant metastases within 2.7 years, with clinical diagnosis occurring 18–20 years after the genesis of the founder cell. *Jones et al*. also reported that a benign colorectal tumor might require 17 years to develop into an advanced carcinoma [56]. On a larger timescale, *Meza et al*. reported that the average time from an initial premalignant mutation to the ultimate conversion of a detectable cancer in pancreatic and colorectal cancers may take up to 50 years [54]. While tumor progression timelines may vary for different cancers, these studies share the implications that a period of at least 20 years since inception of the first malignant cell should pass before a primary tumor becomes detectable.

Our modeling results can also be correlated with published comprehensive genomic studies of clinically annotated HGSOC samples. For example, The Cancer Genome Atlas Research Network examined 489 HGSOC tumor samples, and provided the most comprehensive and integrated catalogue of (epi)genomic changes associated with HGSOC progression to date [57]. An outcome of our model is that an estimated 5 to 8 infrequent, rate-limiting events associated with changes in the initial growth saturation rate, *k*_*decay*_ are required to reach a baseline TVU detectable or life-threatening untreated HGSOC tumor volume. Additionally, we note the substantial heterogeneity in the number of such genomic aberrations predicted by our model, and observe that on average, one or two *extra* events are required in order for a detectable HGSOC tumor volume to become life-threatening. Our modeling findings align with the reported heterogeneity and number of the HGSOC-associated pathways altered in clinical HGSOC samples, as identified in [57] (see Fig 3 therein).

Our mathematical modeling approach also represents a novel *in silico* framework aimed at modeling HGSOC growth and progression. Surprisingly, few similar mathematical modeling inferences regarding the evolution of ovarian cancers or estimating the efficacy of various ovarian cancer screening strategies have been published to date. Durrett *et al*. developed a multi-type branching processes model for ovarian cancer growth and progression to estimate the window of opportunity for screening, which they define as the time during which TVU-based tumor detection can result in a significantly reduced chance of mortality [30]. Based on their mathematical analysis, it is predicted a window of opportunity of 2.9 years, thus ovarian cancer screening should occur at least biannually. In another example, Brown and Palmer used a Monte Carlo method to fit an exponential *in silico* model for tumor growth, with separate growth rate parameters for early and advanced stage serous ovarian cancers [31]. The Brown and Palmer study was based on occult tumor size data collected from healthy germline *BRCA1* mutation carriers who had their ovaries and Fallopian tubes prophylactically removed. They estimated the window of opportunity for TVU detection of early stage occult serous cancers to be 4.3 years, and predicted that most serous cancers would progress to an advanced stage a median of 0.8 years prior to clinical, surgical detection. Nonetheless, these existing mathematical efforts, conducted towards modeling ovarian carcinogenesis or estimating the efficacy of various ovarian cancer screening strategies, do not properly account for the considerable degree of heterogeneity of the disease [57, 58] and correlate primary tumor size with metastatic potential, disregarding clinically reported findings of low primary tumor volume advanced-stage HGSOCs or large primary tumor volume early-stage HGSOCs [6]. In contrast, our mathematical investigation focuses specifically on modeling HGSOC growth and progression, and does not link primary tumor volume to metastatic potential. Moreover, our findings show that multiple frequency TVU monitoring across various TVU detection sensitivities does not significantly improve the detection of HGSOC tumor volumes in an *in silico* cancer-positive HGSOC population.

Several limiting assumptions were made in our model. First, we do not distinctly address the underlying mechanism behind either HGSOC initiation or its progression, but it is well known that many factors may contribute to HGSOC carcinogenesis and progression (e.g. loss of function of tumor suppressor gene *p53* and the disruption of the homologous recombination repair pathway via somatic or germline mutations of the *BRCA1* and *BRCA2* genes [11, 57, 58]). Second, we assume that the initiation of HGSOC occurs at some point during a woman’s premenopausal stage, and we increment time in intervals of 28 days (the average length of a menstrual cycle), to reflect subsequent potential changes in the growth saturation rate. A clinically recognized risk factor for HGSOC progression is the number of ovulatory events during a woman’s lifetime [5, 10]. Third, we do not associate a direct cost to a more rapid cell cycle time (or faster doubling time), even though one probably does exist *in vivo*. Given the model sensitivity to initial conditions (the initial tumor growth rates), we chose conservative baseline TVU detection and life-threatening volume thresholds. Variation in the model parameters or baseline thresholds would only result in a faster or delayed HGSOC progression, but would not yield substantially different median or range values for the estimated cdf’s. A reasonable parameter set range would, however, enable us to obtain sharper estimates. Finally, it is possible that HGSOC rates of cellular division may vary within different subcellular populations belonging to the tumor volume. For simplicity, we do not distinguish between the various subpopulation growth rates, as such values are difficult to quantify empirically.

The HGSOC growth and progression model presented here represents an initial and novel attempt to model *in silico* a clinically occult pathological process, and obtain quantitative estimates of otherwise unknown statistics that are impossible to obtain even in large-scale prospective cohort screening studies (i.e. time needed to reach baseline TVU detectability, time needed to reach baseline life-threatening untreated tumor volume, and window of opportunity interval length). Our mathematical model provides a quantitative mathematical explanation that supports clinical findings such as the ones reported in [40] and results from prospective TVU screening trials such as the UKTOCS or PLCO, and thus represents a novel attempt to explain why multiple, large-scale TVU-based HGSOC detection screening studies have not proven significant mortality benefits. Our model is consistent with case reports and prospective TVU screening population studies in that a key prediction of our model is that HGSOC detection is not amenable to frequent TVU monitoring. The mathematical model provides support to the empirical recommendation against frequent HGSOC monitoring or screening [25].

## Supporting Information

S1 File **Fig A.** Workflow behind the HGSOC growth model. **Table A.** The baseline parameter values used in the model simulations. **Table B.** Cell-number-to-volume and tumor diameter conversion. **Table C.** Statistics generated from one sample simulation of the HGSOC growth and progression model illustrating the time needed to reach the baseline TVU detection threshold, the baseline life-threatening tumor volume (TLV), the window of opportunity interval length (WOP), and the number of HGSOC growth curves that never reach TVU baseline detectability (occult), or the life-threatening threshold (regressed) volumes, respectively, during the sample simulation. **Table D.** The data used to produce Fig 6A and 6B.
(DOCX)
