# Supplementary material for: Modeling the Dynamics of High-Grade Serous Ovarian Cancer Progression for Transvaginal Ultrasound-Based Screening and Early Detection
Source: PLoS One. 2016 Jun 3;11(6):e0156661. doi: 10.1371/journal.pone.0156661 (PMC4892570; doi:10.1371/journal.pone.0156661)
Supplement: S1 File — Fig A. Workflow behind the HGSOC growth model. Table A. The baseline parameter values used in the model simulations. Table B. Cell-number-to-volume and tumor diameter conversion. Table C. Statistics generated from one sample simulation of the HGSOC growth and progression model illustrating the time needed to reach the baseline TVU detection threshold, the baseline life-threatening tumor volume (TLV), the window of opportunity interval length (WOP), and the number of HGSOC growth curves that never reach TVU baseline detectability (occult), or the life-threatening threshold (regressed) volumes, respectively, during the sample simulation. Table D. The data used to produce Fig 6A and 6B. (DOCX) [file pone.0156661.s001.docx]

**Supporting Information**

Since baseline abnormal ovarian volumes were not reported in [1] we chose to define abnormal, cancer-positive ovarian enlargement as two standard deviations above normal ovarian volume, using the values 20$cm^{3}$ and 10$cm^{3}$ for pre- and postmenopausal women, respectively, as reported in [2]. We assumed all the patients younger than 50 years of age reported in were premenopausal, and conversely, that all patients older than 50 were postmenopausal. Herein, we define menopause as occurring 12 months after a patient’s last menstrual cycle and confirmed by follicle stimulating hormone levels > 40 IU/L [3]. Using the above definitions for abnormal ovarian volumes stratified by menopausal stages and the assumption that all abnormal ovarian volumes reported are also HGSOC-positive, we obtain conservative lower bounds for the initial HGSOC growth rate in the *in silico* cancer-positive population.

**S1 Fig A. Workflow behind the HGSOC growth model.**

Changes are globally implemented, meaning that once a stochastic jump in $k_{decay}$ occurs, cells proliferate according to the newly updated Gompertz-type growth law. We stop all simulations when the time since the inception of the first HGSOC cell (t) reaches 38.5 years, which for a premenopausal case, is equivalent to an average of 460 menstrual cycles a woman with two full-term pregnancies experiences; if the respective HGSOC growth curve reaches TVU detectability, we compute the time since the inception of the first HGSOC cell until clinical detection is reached. Similarly we compute the time until clinical life-threatening HGSOC tumor volume is reached if the respective HGSOC growth curve reaches that stage. In the simulations performed the initial $k_{growth}$ is uniformly sampled from the values in Fig 1.

We set the initial $k_{\mathrm{decay}}$to be initial $\frac{k_{\mathrm{growth}}}{2}$ and implement the changes in the initial growth saturation rate,$k_{\mathrm{decay}}$, in a two-step manner: first, we generate$\alpha\sim\ln N({10}^{-2}, 25\cdot{10}^{-2})$, that is log-normally distributed with mean = 10^-2^ and variance = 25 x 10^-2^ and check if α is less than a randomly generated number between 0 and 1. If that is the case, we generate another random number between 0 and 1 and compute the updated $k_{\mathrm{decay}}$as$\frac{\mathrm{previous}k_{\mathrm{decay}}}{1 + random number}$. We then allow the number of HGSOC cells, N(t), to follow the Gompertzian growth law until the probability of a random change in $k_{\mathrm{decay}}$ occurs again, which leads to another update. Computations of the simulated growth curves are performed until the simulation time runs out, that is, when the time since the inception of the first HGSOC cell reaches 38.5 years; we do this for each of the *n = 1000* simulated growth curves.

The initial HGSOC growth rate values and range, $k_{\mathrm{growth}},$ are as reported in Table A in S1.

**S1 Table A. The baseline parameter values used in the model simulations.**

| **Parameter** | **Description** | **Value** | **Unit** | **Simulated Range** | **Source** |
| --- | --- | --- | --- | --- | --- |
| $k_{\mathrm{growth}}$ | Initial HGSOC growth rate | Median = 0.0133 (*) | day^-1^ | 0.0014- 0.0448 | Estimate based on [1] |
| $k_{\mathrm{decay}}$ | Initial HGSOC growth saturation rate | $\frac{k_{\mathrm{growth}}}{2}$ | day^-1^ | – | Initial estimate based on [1] (**) |
| $N_{0}$ | Initial, pre-diagnosis HGSOC cell count | 1 | cell | – | – |
| α | Probability of random change in$k_{\mathrm{decay}}$ | Median = 0.015 (**) | – | $\alpha\sim ln N({10}^{-2}, 25\cdot{10}^{-2})$  range =$0.0094 -0.150$ | – |

(*) The convention for the cell number-to-volume conversion used is 1 cm^3^=1 cc=10^9^ cells [4].

(**) Values are subsequently updated according to the algorithm illustrated in Fig A in S1.

**S1 Table B. Cell-number-to-volume and tumor diameter conversion.**

| **Volume (cm^3^)** | **Cell-number count (cells)** | **Equivalent spherical tumor diameter (cm)** |
| --- | --- | --- |
| 0.5 | 0.5 x 10^9^ | 0.98 |
| 1 | 10^9^ | 1.24 |
| 1.5 | 1.5 | 1.42 |
| 10 | 10^10^ | 2.67 |
| 20 | 2 x 10^10^ | 3.36 |
| 1000 | 10^12^ | 12.4 |

**S1 Table C. Statistics generated from one sample simulation of the HGSOC growth and progression model illustrating the time needed to reach the baseline TVU detection threshold, the baseline life-threatening tumor volume (TLV), the duration of the window of opportunity interval (WOP), and the number of HGSOC growth curves that never reach TVU baseline detectability (occult), or the life-threatening threshold (regressed) volumes, respectively, during the sample simulation.**

Baseline parameters used to simulate *n = 1000* HGSOC growth curves are as specified in Table A in S1. Here, the following definitions are used: TD = min {t ≥ 0 such that N(t)=10^10^ cells$t \geq0 such that N\left( t \right) ={10}^{10}\mathrm{cells}$}, TLV = min{t ≥ 0 such that N(t)=10^12^ cells$t \geq0 such that N\left( t \right) ={10}^{12}\mathrm{cells}$}, WOP = TLV – TD, *occult* represents the number of HGSOC growth curves that never reach the detectability threshold (i.e. TD = 0), and *regressed* represents the number of HGSOC growth curves that never reach the life-threatening size threshold (i.e. TLV = 0). Cell-number-to-volume and tumor diameter conversions are reported in Table B in S1.

The number of HGSOC growth curves that never become detectable (*n = 509*), and life-threatening, respectively (*n = 582*) are subtracted from *n = 1000* simulated HGSOC growth curves in the monitoring frequency analysis. Notice that it takes approximately 26 years for a representative HGSOC growth curve to become detectable, and about 27 years for an untreated HGSOC growth curve to become life-threatening. The expected window of opportunity interval length for curves that reach both thresholds is expected to be concentrated around 1.8 years.

| **Statistic** | **TD (years)** | **TLV (years)** | **WOP (years)** |
| --- | --- | --- | --- |
| Median | 25.85 | 27.3 | 1.76 |
| Mean | 25.7 | 26.73 | 2.6 |
| STD | 7.94 | 7.4 | 2.85 |
| Min. | 4.14 | 4.75 | 0.3 |
| Max | 38.43 | 38.6 | 23.47 |

**S1 Table D. The data used to produce Fig 6A–B.** Each table entry represents the difference between consecutive screening frequencies (e.g. 79, the leftmost entry, bottom row, represents the number of additional HGSOC growth curves that would be missed when switching from a 4-year monitoring frequency to a 5-year monitoring frequency) or consecutive TVU detectability sensitivities (e.g. 36, the second leftmost entry, bottom row, represents the number of additional HGSOC growth curves that would be missed when switching from a 1cm^3^ TVU detection threshold to a 1.5cm^3^ TVU detection threshold).

| **Monitoring frequency** | **TVU sensitivity** | | | |
| --- | --- | --- | --- | --- |
|  | **10 cm^3^** | **1.5 cm^3^** | **1 cm^3^** | **0.5 cm^3^** |
| 6 months | 9 | 0 | 0 | 0 |
| 1 year | 26 | 13 | 3 | 0 |
| 2 years | 61 | 34 | 23 | 12 |
| 3 years | 70 | 29 | 37 | 25 |
| 4 years | 78 | 33 | 46 | 50 |
| 5 years | 79 | 36 | 60 | 71 |

Bibliography

1. Horiuchi A, Itoh K, Shimizu M, Nakai I, Yamazaki T, Kimura K, et al. Toward understanding the natural history of ovarian carcinoma development: a clinicopathological approach. Gynecol Oncol. 2003;88(3):309-17.

2. Pavlik EJ, DePriest PD, Gallion HH, Ueland FR, Reedy MB, Kryscio RJ, et al. Ovarian volume related to age. Gynecol Oncol. 2000;77(3):410-2.

3. Harlow SD, Gass M, Hall JE, Lobo R, Maki P, Rebar RW, et al. Executive summary of the Stages of Reproductive Aging Workshop + 10: addressing the unfinished agenda of staging reproductive aging. J Clin Endocrinol Metab. 2012;97(4):1159-68.

4. Chignola R, Foroni RI. Estimating the growth kinetics of experimental tumors from as few as two determinations of tumor size: implications for clinical oncology. IEEE Trans Biomed Eng. 2005;52(5):808-15.
